# Progress and Challenges in Industrially Promising Chemical Vapour Deposition Processes for the Synthesis of Large-Area Metal Oxide Electrode Materials Designed for Aqueous Battery Systems

**DOI:** 10.3390/ma14154177

**Published:** 2021-07-27

**Authors:** Dimitra Vernardou

**Affiliations:** 1 Department of Electrical and Computer Engineering, School of Engineering, Hellenic Mediterranean University, 71410 Heraklion, Greece; dvernardou@hmu.gr; Tel.: +30-2810-379631; 2 Institute of Emerging Technologies, Hellenic Mediterranean University Center, 71410 Heraklion, Greece

**Keywords:** CVD, large-area manufacturing, electrodes, electrochemical performance

## Abstract

The goal of the battery research community is to reach sustainable batteries with high performance, meaning energy and power densities close to the theoretical limits, excellent stability, high safety, and scalability to enable the large-scale production of batteries at a competitive cost. In that perspective, chemical vapour deposition processes, which can operate safely under high-volume conditions at relatively low cost, should allow aqueous batteries to become leading candidates for energy storage applications. Research interest and developments in aqueous battery technologies have significantly increased the last five years, including monovalent (Li^+^, Na^+^, K^+^) and multivalent systems (Mg^2+^, Zn^2+^, Al^3+^). However, their large-scale production is still somewhat inhibited, since it is not possible to get electrodes with robust properties that yield optimum performance of the electrodes per surface area. In this review paper, we present the progress and challenges in the growth of electrodes through chemical vapour deposition at atmospheric pressure, which is one procedure that is proven to be industrially competitive. As battery systems attract the attention of many researchers, this review article might help those who work on large-scale electrical energy storage.

## 1. Introduction

**Figure 1:**
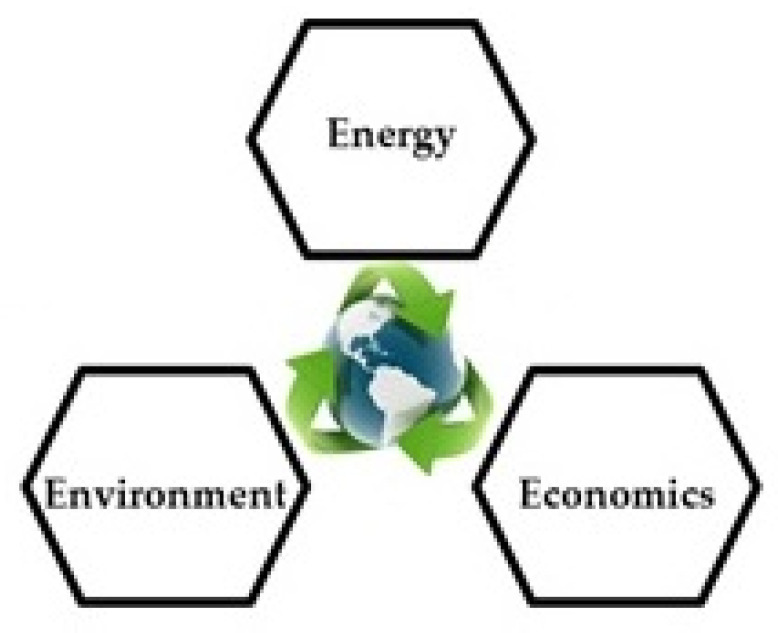
Challenge for technology innovation, integration, and efficiency of usage through the balance estimation among energy, the environment, and the economy.

Due to the ever-increasing need for less dependence on fossil fuels, there is both a societal and technological drive towards environmentally friendly and longer-lasting energy technologies. Renewable energy technologies such as solar and wind are already reducing the usage of fossil fuels by two-thirds, and by 2030, the generation from renewable sources is expected to be cheaper [1]. Nevertheless, versatile and economically desirable forms of energy storage are needed to provide the energy required during the high demand times. From that perspective, the challenge for technology innovation, system integration, and efficiency is the balance estimation among energy, the environment, and the economy, as indicated in Figure 1.

The energy storage market can be divided into two sectors: the stationary and the mobile [1]. The stationary sector is related with the cost per unit energy, while the mobile sector is expressed as cost per unit energy density. Specifically, energy industry applications including electric vehicles, and large-scale energy storage systems (EESs) require EESs with safety, high reliability, advanced energy density, and low cost [2]. This means that inexpensive materials as well as low energy- and time-consuming processes will be needed to meet the performance targets [3,4].

Of particular interest, the most studied systems feature lithium-ion batteries [5,6]. Despite the high energy densities possible with these storage systems, safety remains an issue in security-critical applications as highlighted by incidents such as the Boeing 787 battery fires in 2013, Samsung Note 7 explosions in 2016, and Tesla Model S combustions in 2019 [7]. Additionally, the low abundance of lithium has given rise to the development of alternative cations [8]. Sodium-ion batteries are the closest technology to today’s lithium-ion batteries, but their low specific capacity creates an obstacle for their utilisation [9]. Potassium-ion batteries are a good option, but they are still at the early stages of development [10]. Potassium presents slow diffusion kinetics in solids and yields in lower gravimetric capacities than sodium [9]. On the other hand, magnesium-ion batteries are reactive enough to become deactivated over time due to the strong tendency of magnesium anodes to form passivating surface films. This can be solved through the combination of magnesium with different cathodes to provide good specific capacities, but with a lower voltage compared with lithium-ion batteries (<1.3 V, compared with 3.8 V for Li-ion batteries) [9]. In terms of zinc metal anodes, their performance is hindered by the zinc dendrite formation and side reactions occurring on the anode including the corrosion [11]. Finally, aluminium is the most promising alternative thanks to its three–electron redox reaction. Nevertheless, there are stability issues to consider when using Al [12].

**Figure 2:**
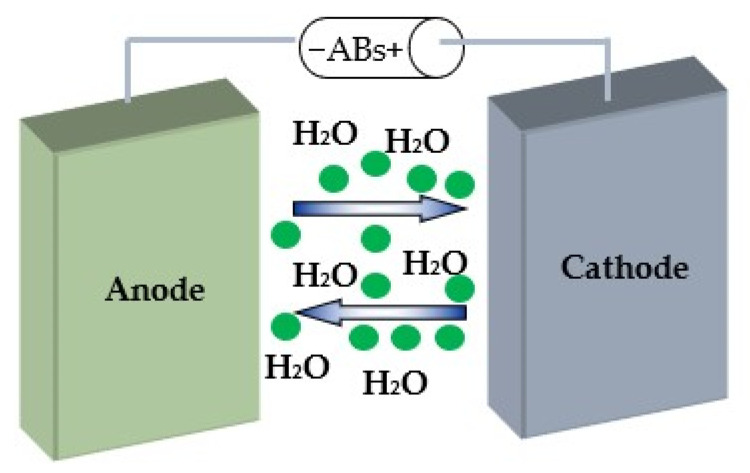
Schematic presentation of aqueous batteries. The anode and cathode are separated by a membrane filled with monovalent or multivalent ions (green circles) in aqueous solution.

Aqueous-based batteries (ABs) are promising solutions for large-scale applications, since a water-based battery system can be produced at relatively low cost, while they are also generally safe to use and require no use of inert atmospheres during production. Figure 2 presents a schematic presentation of an aqueous battery indicating the negative electrode called anode and the positive electrode called cathode. The anode and cathode are separated by a membrane filled with electrolyte. The key difference between conventional and flow batteries is how energy is stored. Conventional batteries store energy in electrodes, while flow cells store it in electrolytes.

In addition, such systems are environmentally benign and scalable, yielding high power densities, while also being quite tolerant towards mishandling [13,14,15,16]. Up to the present, various types of materials have been developed, which show long cyclability and high rate capability for aqueous battery systems [12,17,18,19]. However, there is a drawback if one considers the voltages that are able to be achieved. The electrochemical window cannot be extended above 1.23 V, since water begins to “split” into hydrogen and oxygen gases. However, a highly concentrated aqueous electrolyte has enabled 100% Zn plating/stripping efficiency [20]. In addition, the introduction of surfactant materials into the electrolyte could be beneficial in terms of inhibiting the decomposition of water, suppressing the corrosion of metal anode, and enlarging the stability window of aqueous electrolytes [21].

The major electrode components can be divided into three main categories. The first one is carbon materials [22]. Graphene and chemically modified graphene sheets have shown a high electrical conductivity, high surface area, and good mechanical properties comparable with, or even better than carbon nanotubes [23,24]. The second category is the transition metal oxides including MnO_2_ and V_2_O_5_, which are used for the fast and reversible redox reactions at the surface of the active material [25,26]. However, they usually have a high electrical resistance, resulting in a low power density. The third one is related with the conductive polymers, which suffer from a limited stability during cycling that reduces the performance when used as bulk materials [27].

Among the possible electrode materials, metal oxides such as V_2_O_5_, TiO_2_, and MnO_2_ have attracted a lot of interest due to their ability to form high-energy density structures, their relatively high earth abundance, and lack of any major safety or environmental issues associated with their use. The decrease of metal oxide particle size to the nanoscale level is assumed to result in larger electrode/electrolyte contact areas and the ability to more effectively accommodate the strain of cation intercalation/deintercalation in nanomaterials [6]. Although they have been optimised to meet the requirements of portable electronics, certain intrinsic characteristics such as fabrication cost and safety in terms of organic electrolytes utilised prevent their use as viable large-scale ESSs [23]. The necessity of creating robust and sustainable batteries is the dominant factor of the battery community, which would eventually lead to the next generation of electronics for a “greener” and ultimately better future.

Towards this direction, the battery engineers have turned their attention to fabrication process and materials [1]. On the one hand, an expensive fabrication process needs to be avoided for the engineering of low-cost battery cells. On the other hand, materials that require a high production cost need to become economically attractive in the coming years.

As a consequence, the development of a low-cost and eco-friendly manufacturing process to fabricate batteries is paramount. The US Department of Energy suggests that energy storage systems must meet a cost target of $125 per kWh^−1^ to meet the requirements for widespread adaption, which require a three- to four-fold reduction in system costs [28]. Therefore, we need to underline that the most crucial factor for any material to be commercially viable for industrial-scale application is its cost-effectiveness.

From that perspective, chemical vapour deposition (CVD) offers a balance between efficiency, controllability, tunability, and excellent run-to-run repeatability in the coverage of monolayer on an adequate substrate at low operational and maintenance cost. Especially, when performed at atmospheric pressure, it is compatible with in-line manufacturing properties. Additionally, the material properties can be controlled with great accuracy by varying growth parameters such as temperature, precursor composition, and flow rate giving unique morphologies, which may then be further tuned if a solvent-based aerosol CVD process is used by varying the solvent. The importance and significance of CVD manufacturing processes are further discussed in the following sections.

In this review paper, special emphasis will be given to the connection between advanced materials and CVD for Li, Na, K, Zn, Mg, and Al battery systems, following the literature published in the last five years.

## 2. Manufacturing Technologies

In terms of the mass production of materials, the main factors to consider are the production cost, scalability, reproducibility, processability, and the quality of the final products. Several strategies have been developed for the electrode manufacture including both wet and dry processes.

### 2.1. Wet Chemistries

Generally, wet-chemistry strategies are utilised for the fabrication of a broad range of materials and composites since they are simple and easily operated. Nevertheless, there are difficulties in terms of forming continuous films on certain substrates in most of the wet processes [29]. In addition, the roll-to-roll wet processing techniques (i.e., the slurry method) require the rigorous mixing of a highly toxic and flammable solvent solution, the casting into metallic collectors, and finally the drying procedure. From that perspective, the solvent evaporation is a high-cost industrial processing step for electrode fabrication, which adds both additional energy and time inputs into the electrode fabrication process [30,31].

Regarding the spin-coating, it is a process that is utilised extensively to deposit uniform coatings of organic materials on flat surfaces [32]. The procedure is typically divided into four steps including the deposition, spin up, spin off, and evaporation. Evaporation of the solvent is possible during the rapid rotation. However, the highly volatile components require drying of the applied layer in order to evaporate. In addition, the size of the substrate is an obstacle. In this case, high-speed spinning is required, which is difficult to achieve, and as a consequence, the material efficiency is very low.

Spray pyrolysis methods are based on the formation of an aerosol from various precursor solutions. This method typically involves the following steps [33]:

1. Evaporation of the solvent.
2. Drying the droplets containing the precipitated solute.
3. Annealing of the precipitate at high temperatures.
4. Formation of microporous particles.
5. Formation of solid particles.
6. Sintering of solid particles.

Compared with other procedures, this approach has advantages including the simple equipment and experimental arrangement making it a cost-effective method. Nevertheless, it is sometimes difficult to determine the optimum growth temperature for a large-area substrate surface.

Sol–gel processes are wet chemical techniques for the production of solid inorganic materials and involve a number of reactions including the hydrolysis and polycondesation of precursors that are either metal alkoxides or inorganic metal salts such as sulphates, nitrates, and chlorides. The principal disadvantage of this procedure is the removal of the organic groups from materials. In this case, slow drying along with annealing is often required, which sometimes results in nanoparticles aggregation, restricting their potential applications [33].

The combination of a hydrothermal and microwave-assisted synthetic procedure is capable of reducing the growth temperature and time and as a consequence the consumption of electricity and the overall costs of chemical synthesis [34]. Considering the growth of materials in short time intervals and the higher product yield as compared with the conventional synthetic methods, this technique is indeed environmentally friendly [35]. Nevertheless, it is difficult to control the particle size, purity, and morphology of material at large scale (i.e., larger equipment and solvent amounts are required).

In all cases, the controllability of the interface between two materials (i.e., for instance graphene and metal oxides) for battery applications remains a challenge to overcome. This is because they are simply synthesised by mixing or dispersing their components (i.e., inorganic components with graphene), which however leads to a poor interfacial interaction. Hence, a good understanding of the materials’ surface chemistry is important for increasing interfacial interaction and achieving a well-defined structure of the electrode. In general, through these routes, the precise formation of well-defined structures is possible but with low productivity [36].

### 2.2. Dry Processes

Moving toward the dry electrode coating techniques, these can reduce the cost and the environmental impact of the batteries production since they come with many benefits. In particular, the energy-intensive drying process is entirely avoided, lowering the capital cost requirements and allowing similar or even enhanced performance compared with other coating techniques [37,38].

Additive manufacturing techniques such as 3D printing have directed attention to the large-scale development of components for energy applications [39], since it is an inexpensive and simple fabrication method that relies on media characteristics and a three-axis motion stage to create particular structural forms [40]. In this method, it is necessary to utilise materials with excellent rheological properties when dispersed in solvents such as water, which is a limitation regarding the range of materials that can be developed.

CVD is one process that is utilised to form a thin layer of material onto a substrate. Vapour deposition routes are the preferred processes for the formation of products with superior hardness and oxidation resistance. The basic principles of CVD operation with the respective advantages and disadvantages are discussed in the following sections.

CVD is based on the principle of adding a new layer of material onto a substrate surface and therefore belongs to the additive manufacturing technique family. CVD routes do not require any external driver for the solidification process such as the spread binders in 3D printing. In particular, it is a route for depositing a material in a controllable manner via the thermal decomposition of precursors [41]. A chemical reaction is initiated in the chamber between the decomposed products and a substrate that causes the precursor gas to react or break down into the desired solid material and bond to the substrate surface in the form of a thin film [42]. By varying experimental processing parameters such as substrate material, substrate temperature, reaction gas mixture, precursors, and total pressure gas flows, materials with a wide range of properties can be grown [43,44,45]. CVD techniques enable the production of coatings with uniform thickness and low porosity even on substrates with complicated shape and patterned surfaces [46]. The main CVD processes are summarised below. The first set of processes is classified by the operating pressure:

- Atmospheric pressure CVD (APCVD): Processes at atmospheric pressure/Precursors are either in liquid or powder form/Precursor vapours are transported in the reaction chamber by means of inert gases.
- Low-pressure CVD: Processes at sub-atmospheric pressure/Reduced pressures tend to eliminate unwanted gas-phase reactions and improve film uniformity across the substrate surface/Similar precursor characteristics with APCVD.
- Ultra-high vacuum CVD: Processes at very low pressure (<10^−6^ Pa)/Similar precursor characteristics with APCVD.
- In addition, they can be divided by the physical characteristics of vapour:
- Aerosol-assisted CVD (AACVD): Similar precursor characteristics with APCVD/Precursors are transported to the substrate by means of an aerosol. In this route, the precursors are non-volatile.
- Direct liquid injection CVD: Precursors are solid dissolved in convenient solvent, which are injected in the chamber towards injectors/Precursor vapours are transported to the substrate as in APCVD process/High growth rates can be achieved.
- Furthermore, there are the plasma methods, which include the following:
- Plasma-enhanced CVD: Plasma is utilised to enhance the chemical reaction rates of the precursors/Lower growth temperatures (even at 120 °C) than APCVD are allowed.
- Other procedures include the following:
- Hot wire CVD: Hot filament is utilised to chemically decompose the source gas/Difficult to control the substrate temperature for a large area, since an increase in catalytic surface requires a proportionally larger supply of source gases.
- Initiated CVD: Production of polymeric thin films using one or more monomer species along with initiator/Higher deposition rate and energy required as compared with hot wire CVD due to the free-radical initiating species/Highly desirable for the surface modification of thermally sensitive substrates/Low vacuum required in the range 13 to 133 Pa.
- Atomic layer deposition (ALD): Layers of different materials can be produced/Annealing is often required to get crystalline materials/Excellent control of thickness and uniformity/ALD proceeds through two half-reactions, i.e., the one after the other in contrast with the CVD, which is a continuous process; i.e., all reactants are supplied at the same time and in a faster rate than ALD.

CVD has numerous advantages [47], as shown below:

(1) It is a non-line-of-sight process, which leads to the good conformality in terms of uniform thickness of the coating.
(2) Various range of precursors such as halides, hydrides, and organometallic compounds can be utilised, enabling the deposition of a large spectrum of materials.
(3) The growth temperature can range from 1000 °C to around 125 °C or less.
(4) Excellent control over the crystal structure, stoichiometry, surface morphology, and orientation of the final materials through the precise control of the processing parameters.
(5) The deposition rate can be adjusted depending on properties needed for the particular application.
(6) CVD operated at atmospheric pressure working environments is a viable solution for industrial-scale deposition.
(7) The flexibility of CVD processes variations is advantageous because it allows many changes in composition. Deposition of layers and composites can be readily achieved.

Nevertheless, it has also several disadvantages

(a) In some cases, it requires chemical precursors that present safety and health hazards such as SiCl_4_ and B_2_H_6_. In addition, the exhaust gases consist of by-products and intermediates such as CO, H_2_, or HCl, which can be hazardous, toxic, corrosive, and flammable in high concentrations when they are released into the environment.
(b) It has a sort of complexity because numerous runs are required to determine and reach the suitable growth parameters. This can be overcome through computational fluid dynamics (CFD) simulations to eliminate unexpected deformation of the coated surface, since it can be performed to evaluate the whole experimental process, before, during, and after the experimental procedure.

### 2.3. Progress in the Development of Aqueous Batteries

The progress of aqueous battery systems has proceeded at a rapid pace and has included studies of monovalent Li^+^, Na^+^, K^+^, divalent Mg^2+^, Zn^2+^ and trivalent systems Al^3+^. These aqueous systems show enhanced energy density and stability compared with acidic and alkaline systems, since the structure does not change significantly upon the cation intercalation/deintercalation processes [48]. Among these systems, Li-based aqueous batteries are the most studied due to their benefits in terms of cost and capability performance [49]. Nevertheless, lithium supplies are limited forever, and promising alternatives are related with the sodium and potassium because they are more abundant than lithium [9]. Their ionic radius (Table 1 [50]) is larger than lithium, limiting the range of compounds that can be utilised. The ionic radius is tightly associated with the electrochemical performance of the battery.

Multivalent ion batteries such as Mg^2+^, Zn^2+^, and Al^3+^ have attracted interest from researchers over the last few years due to the increased energy density arising from the presence of high-capacity multivalent metals as anodes. When comparing the monovalent with the multivalent systems, the attained capacity is higher for the multivalent systems due to the fact that multivalent ions can transfer two or three electrons per ion [51].

The magnesium ion battery is arguably the most explored multivalent battery system due to its lower price, high volumetric capacity, and abundance (i.e., the eighth most abundant element in the earth’s crust) [52]. Despite the fact that its ionic radius is similar to lithium, magnesium ions have high charge density, which results in a strong polarization effect and sluggish diffusion process in cathode materials. On the other hand, aqueous zinc ion batteries are gaining a lot of attention due to the high abundance and capacity of Zn metal along with the excellent electrochemical stability in water [53]. Aluminium is also considered an attractive option for batteries due to its high abundance, high gravimetric density, lower reactivity, and easier handling [12]. It can theoretically provide three times more charge per transferred ion as compared to lithium due to its trivalence. However, an insufficient cycle life and low capacity rate limit its usage [12]. In general, the high charge density of the multivalent ions results in numerous interactions within the lattice of the host intercalation material and with the other species present in the electrolyte, making the diffusion of the high charge-density ion slow [54,55]. How much more this can be affected if one considers a large area electrode is a big question. In these systems, a major obstacle is the lack of suitable cathode materials. The scarcity of current potential cathode materials is probably due to the lack of knowledge, since for example Zn^2+^ and Al^3+^ have not been among the common cations studied in large-area energy storage systems (ESSs). If successful, multivalent systems can make a major advancement of our understanding of to how to store energy.

In the following sections, we will solely focus on the most recent developments of metal oxide electrodes grown by CVD at atmospheric pressure, used for monovalent and multivalent systems. We will try to give a view on growth challenges and strategies developed to tackle these systems.

## 3. Advances in Aqueous Batteries through CVD Processes

### 3.1. Monovalent Systems

Fluidization thermal CVD (FTCVD) routes in a kilogram-scale fluidised bed CVD reactor was utilised to grow SiO/graphite@C. This method is suitable to uniformly cover the SiO by the carbon layer, avoid particle aggregation and synthesise 1000 g SiO/graphite@C composite per batch using a small amount of methane [56]. In a typical experiment, 300 g SiO was mixed with 700 g graphite particles for 2 h in mixer. Following this procedure, the mixture was loaded in the FTCVD at 1000 °C for 2 h at a heating speed of 10 °C min^−1^ in Ar atmosphere. Electrochemical tests performed in CR2032-type coin-type half batteries showed that the capacity retention of the composite (93.7%) was higher than that of the SiO (55%). The excellent properties of the composite were due to the graphite addition and carbon coating, which served as a buffer to the volume change of SiO, shortened transfer pathway, and improved diffusion coefficient of Li^+^. Electrochemical cycling evaluation of the cell was performed on a Neware battery testing system for a scanning voltage range of 0.01 to 2.0 V.

In an FTCVD reactor, there is a high degree of contact between gases, powders, and reactor walls leading to high heat transfer rates and providing isothermal conditions radially and axially in the bed [57]. This does not happen in the classical CVD processes. Nevertheless, the powder-based electrode material is not free of additives and binders.

To overcome the issues raised by powders, APCVD is proposed for the thin film deposition of vanadium dioxide as electrode. This approach can avoid toxic binders and assure an extensive contact between the active material and the substrate. The contact is much better than that of powder active materials.

**Figure 3:**
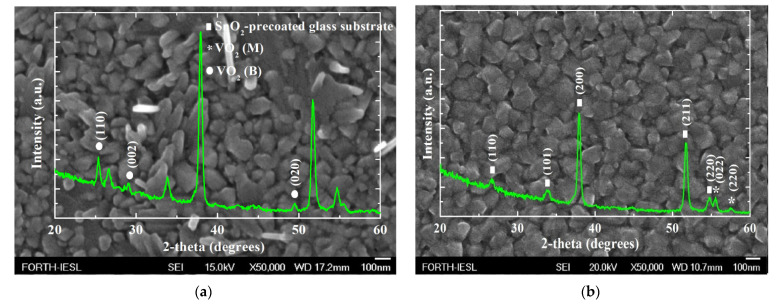
(**a**) X-ray diffraction (XRD) patterns of the APCVD vanadium oxides at 500 °C for 1 L min^−1^ and (**b**) 2.2 L min^−1^ N_2_ flow rate through the VO(acac)_2_ bubbler overlapped on FE-SEM images for ×50,000 magnification [60].

Vanadium dioxide (VO_2_) is a material that has been found to have better performance compared to the well-known V_2_O_5_. This was explained by reference to the higher electronic conductivity and the structural stability arising from the resistance to lattice shearing during cycling [58]. Among the various VO_2_ phases, the metastable phase (VO_2_(B)) is of great interest owing to its layered structure [59]. It was possible to grow vanadium oxides by APCVD at 500 °C using different N_2_ flow rates through the vanadyl (IV) acetylacetonate (VO(acac)_2_) bubbler [60]. The XRD patterns indicated the presence of a VO_2_ (022)-oriented single phase for the highest N_2_ flow rate (i.e., 2.2 L min^−1^) (Figure 3b) [61]. Similar peaks also appeared for the lowest N_2_ flow rate (i.e., 1 L min^−1^), but the peaks were broader and with lower intensity, along with new peaks at 25.4°, 29.2°, and 49.6° (Figure 3a). The three new peaks were attributed to VO_2_ (B), which was the predominant phase. In addition, for the case of 1 L min^−1^, nanocrystallites and outgrowths on the coating surface were indicated, while for the 2.2 L min^−1^, nanocrystallites were predominant (Figure 3).

The electrochemical performance of the samples was evaluated in a three-electrode electrochemical cell as described in [60] utilising Ag/AgCl, Pt, and vanadium oxide (2.5 cm × 2.5 cm × 0.3 cm) as the reference, counter, and working electrode, respectively. The APCVD vanadium oxide for the 1 L min^−1^ N_2_ flow rate presented an enhanced performance due to the co-existence of monoclinic and metastable VO_2_ exhibiting a specific discharge capacity of 425 mAh g^−1^ with capacity retention of 97% after 500 cycles in aqueous Li^+^ electrolyte [60]. In order to understand the significance of the various crystal structures on the performance of the electrodes, in situ Raman analysis with the cyclic voltammetry needed to be performed and established for the future measurements.

Metastable VO_2_ could also be deposited at a lower temperature than before (i.e., 300 °C) utilising vanadyl (V) triisopropoxide (VO(OC_3_H_7_))_3_, which is liquid with high vapour pressure 38.6 Pa at 45 °C, making it highly desirable for handling reasons [61]. In this case, there was no need for an extra precursor for the O_2_ source simplifying the operations. Electrochemical analysis presented a high-performance electrode material for the 2.5 L min^−1^ N_2_ flow rate through the vanadium precursor bubbler, showing a specific discharge capacity of 505 mAh g^−1^ (higher than the theoretical capacity of LiCoO_2_ (140 mAh g^−1^)) with capacity retention of 96% after 500 scans under constant specific current of 1 A g^−1^ [62]. Electrochemical measurements were carried out in a three-electrode cell utilising an aqueous Li^+^ electrolyte and the same set of electrodes as in [60]. This enhanced performance is due to the larger volume of active material available (i.e., highest thickness estimated) as compared with the one reported in [60], making it a promising alternative as a cathode material in Li^+^ batteries if one considers its electrochemical, economic, and environmental merits.

In the system described above with the VO(acac)_2_ precursor [60], the oxygen source could also be altered via the use of propanol and ethanol, indicating the presence of an axis textured VO_2_ monoclinic phase [63]. Electrochemical analysis in an aqueous solution with Li^+^ and vanadium oxide (2.5 cm × 2.5 cm × 0.3 cm) as the working electrode in a three-electrode cell showed promising results for the as-grown sample with propanol, presenting a specific discharge capacity of 459 mAh g^−1^ with capacity retention of 97% after 1000 scans under a specific current of 2 A g^−1^. This performance was due to the higher available active material being less dense at specific sites suggestive of a Stranski–Krastanov type of growth mechanism, as reported in [63].

**Figure 4:**
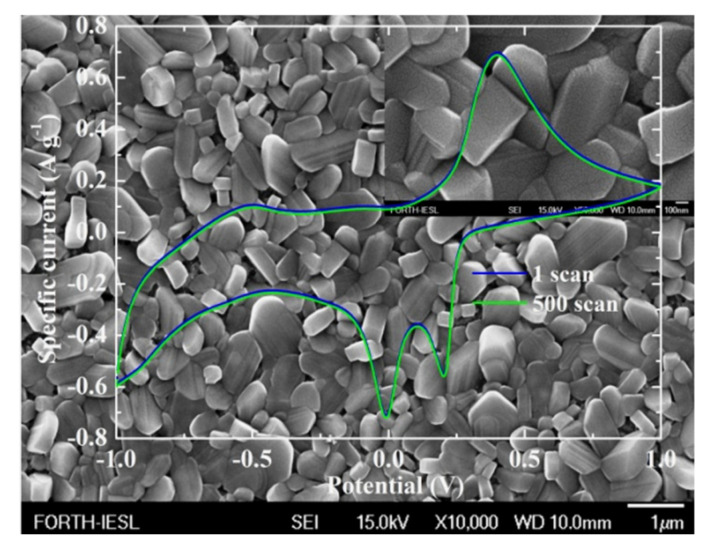
Cyclic voltammetry curves of the first and the 500th scan of the AACVD vanadate bronze for 15% Ag loading at 450 °C on fluorine-doped SnO_2_ precoated glass substrates overlapped on FE-SEM image (FE-SEM image for x50,000 as inset). The scan rate for the cyclic voltammetry curves was 10 mV s^−1^ [66].

Another issue that was considered was the doping for the improvement of the capacitive behaviour [64,65]. In order to study this phenomenon, AACVD was chosen because it has certain advantages over APCVD [66]. These include the single precursor source, which provides good molecular mixing of chemical precursors, enabling the synthesis of multi-component materials with controlled characteristics (structure and morphology), and the compatibility with high-volume manufacturing. In this work, the sample presented rod-like structures, as shown in Figure 4, which showed excellent stability after 500 continuous Li^+^ intercalation/deintercalation scans. The electrochemical cell and the size of the working electrode were the same as those in [60,62,63]. In addition, they showed a specific discharge capacity of 230 mAh g^−1^, which is higher than the nominally pure V_2_O_5_ (22.5 mAh g^−1^). On the basis of this result, AACVD provides a simple and cost-efficient way to grow vanadate bronzes with promising performance as cathodes for Li^+^ batteries.

There has also been considerable attention on the production of flexible energy storage devices due to the demand for flexible, wearable, and implantable electrical devices such as intelligent bracelets and wearable sensors [67,68,69] along with the development of corresponding energy storage systems for power supply. In order to achieve this target, the systems must be non-toxic, light weight, and flexible, with high mechanical strength. Towards this direction, Na^+^ batteries have attracted significant attention. Nevertheless, there are no reports regarding the development and utilisation of metal oxides. It is worth noting that in 2017, Guo et al. [70] demonstrated that belt- and fiber-shaped Na^+^ batteries using 1 M Na_2_SO_4_ aqueous electrolyte displayed promising performance including high power density, high flexibility, and excellent rate capability, and they can be considered suitable for wearable electrical devices. In this work, CVD was carried out to coat carbon on the as-prepared powder of NaTi_2_(PO_4_)_3_. The electrochemical profile of the electrode was investigated by cyclic voltammetry and galvanostatic charge–discharge measurements utilising a three-electrode electrochemical cell with Ag/AgCl and activated carbon film as reference and counter electrode, respectively. The specific capacity was estimated to be 45 mAh g^−1^ in the potential window of 0–0.7 V and 80 mAh g^−1^ for a potential window of 0 to −0.9 V with a capacity retention of 85% after 200 cycles.

### 3.2. Multivalent Systems

**Figure 5:**
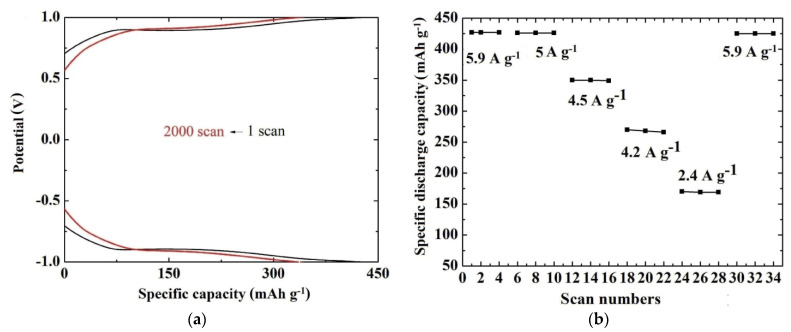
(**a**) Chronopotentiometric curves of the AACVD V_2_O_5_ coating (2 cm × 2 cm × 0.3 cm) at 500 °C at a specific current of 5.9 A g^−1^ for the 1st and the 2000th scan. (**b**) Rate capability of the same sample at specific current values ranging from 5.9 to 2.4 A g^−1^ and then back to 5.9 A g^−1^ [73].

Among the multivalent systems, Mg^2+^ batteries are the most studied [71,72]. As part of these studies, AACVD was performed for the growth of V_2_O_5_ at 500 °C on a fluorine-doped tin dioxide pre-coated glass substrate utilising a solution of vanadium (V) oxytri-isopropoxide in 2-methoxy-ethanol [73]. The as-grown coatings were found to be orthorhombic V_2_O_5_, while the morphology characteristics presented grains that were not uniformly distributed on the substrate [73,74]. The electrochemical studies of the sample were carried out using a three-electrode electrochemical cell [73] in an aqueous solution of 0.075 M, MgCl_2_, which acted as an electrolyte. The current-potential curves indicated an increase of the current with scan numbers showing that the capacity to store Mg^2+^ increases with cycling [73]. The curves remained practically stable after 1000 scans. The specific discharge capacity was estimated to be 427 mAh g^−1^ with capacity retention of 82% after 2000 scans under a specific current of 5.9 A g^−1^ (Figure 5a). The rate capability is also an important parameter for batteries showing good structural stability and high reversibility of the cathode. In this case, it was found that the battery could still deliver a specific discharge capacity of 425 mAh g^−1^ when the specific current was returned to 5.9 A g^−1^, corresponding to a capacity retention of 99% of the original value (Figure 5b).

**Figure 6:**
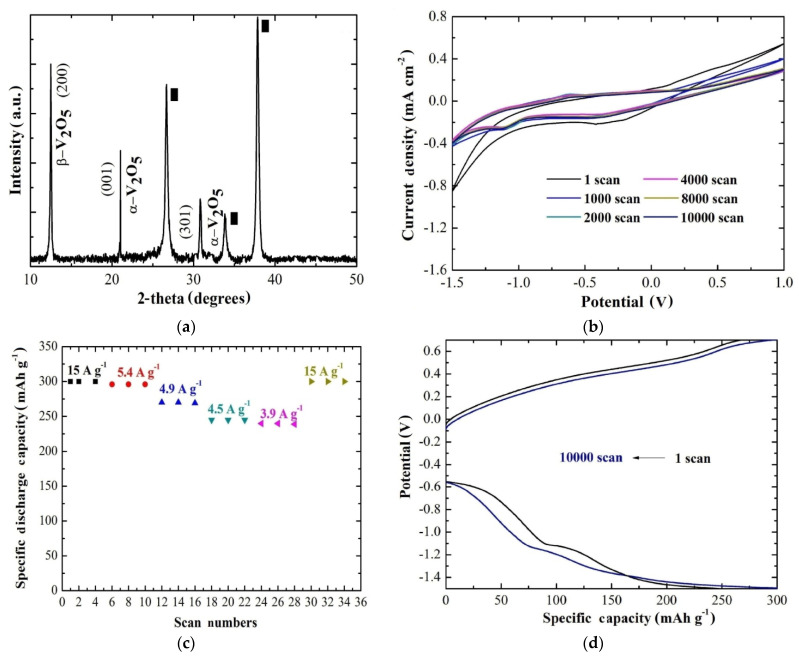
(**a**) XRD pattern of the AACVD V_2_O_5_ coating at 600 °C. (**b**) Cyclic voltammetry curves of AACVD V_2_O_5_ (2 cm × 2 cm × 0.3 cm) at 600 °C after 1, 1000, 2000, 4000, 8000, and 10,000 scans measured in 0.075 M and MgCl_2_ for a scan rate of 10 mV s^−1^. (**c**) Rate capability of the same sample as in (**b**) at different specific current values ranging from 15 to 3.9 A g^−1^ and then back to 15 A g^−1^. (**d**) Chronopotentiometric curves for the same sample as before at a specific current of 15 A g^−1^ for the 1st and the 10000th scan [75].

This was basically the first report showing the viability of AACVD technology to grow V_2_O_5_ cathodes for aqueous Mg^2+^ batteries. This route was further exploited to enhance the stability of the cathode by increasing the substrate temperature to 600 °C [75]. In this way, a mixed phase of orthorhombic and monoclinic V_2_O_5_ was produced, as shown in Figure 6a. The current-potential curves were obtained in the same cell and electrolyte as before, showing an extended stability (Figure 6b) compared with the previous work [73]. In this case, the rate capability of the electrode exhibited a capacity retention of 100% at one order of magnitude higher specific current (15 A g^−1^) compared with the cathode grown at 500 °C (5.9 A g^−1^), as one can see in Figure 6c. Finally, the specific discharge capacity was estimated to be 300 mAh g^−1^ with a capacity retention of 92% after 10,000 scans and coulombic efficiency of 100% (Figure 6d). This enhancement may be the result of a combination of the co-existence of orthorhombic and monoclinic V_2_O_5_ along with the improved adherence of the coating.

In addition to Mg^2+^ batteries, Zn^2+^ has been used in other multivalent ion batteries that have been explored the last few yeas. An important advantage of Zn^2+^ batteries is its potential for wearable applications due to the superior flexibility, adaptability to deformation, and compatibility with the textile industry [76,77]. Nevertheless, there are no reports on CVD metal oxides, as also observed for Na^+^ batteries. In particular, CVD was utilised for the growth of an activated 3D carbon nanotubes (CNT) conductive network followed by the electrodeposition of α-MnO_2_ and finally a layer of PEDOT on the top [77]. In the composite CNT/MnO_2_/PEDOT cathode, homogeneous CNT branches interlinked with each other to form a net-like structure providing a high surface area and favouring the ion/electron movement. The CNT enhanced the kinetics, overcoming the inferior conductivity and mass diffusion rate of α-MnO_2_, while the PEDOT stabilised and prevented the dissolution of the structure during the cycling. Specifically, the cathode delivered a capacity of 306.1 mAh g^−1^ at 1.1 A g^−1^.

If successful, Al^3+^ batteries can make a major advancement on the energy storage industry due to the fact that Al is trivalent and hence is capable of transferring three times the charge achievable with Li^+^. There is research work on CVD for the growth of polymer but not for metal oxides. In the specific research paper, an oxidative CVD (oCVD) process can allow the oxidant and polymer monomer to react and then diffuse to the sample surface for the film formation. It was found that PEDOT on α-MnO_2_/carbon paper showed a high discharge performance with improved conductivity due to the clean charge transfer between PEDOT and α-MnO_2_ as predicted by Density Functional Theory (DFT) study offering a new perspective on the catalytic activity enhancement of Al^3+^ battery [78].

One may conclude from the literature that there are few reports related with the growth of metal oxide electrodes by CVD. This gives room for the optimisation of the CVD process to take over all manufacturing steps of the multi-layers constituting the electrodes as proposed in the following section.

### 3.3. Synopsis

**Figure 7:**
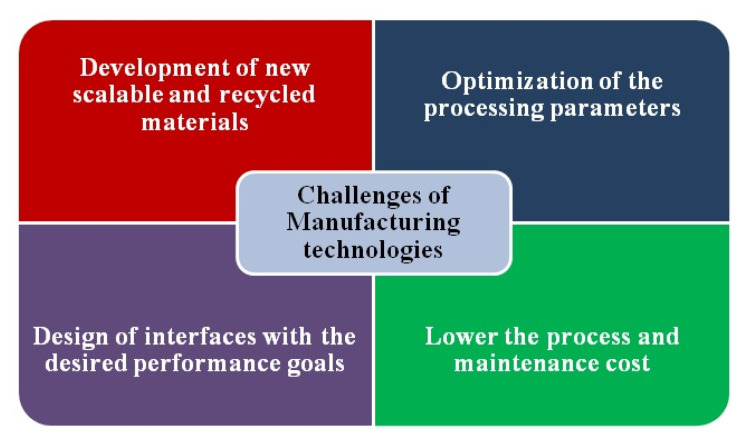
Main challenges encountered in the commercialisation of electrodes.

It has been shown that CVD is a potential technique for the fabrication of electrode materials with good performance in terms of capacity and stability (i.e., almost all electrodes present capacity retention over 80% after thousand of cycles) (Table 2). The main challenges encountered in the commercialisation of electrodes (Figure 7) are as follows:

A. The development of large-area electrodes with high reversibility, high rate capability, and extended cycle stability.
B. The development of new scalable and affordably recycled materials.
C. The optimum interfacial contact of the electrolyte with the electrode material (understanding the mechanisms and improving the properties in small to large scale).
D. The optimisation of the processing parameters through the use of modelling tools to lower the process and maintenance cost.

Combinatorial CVD-based manufacturing processes are promising alternatives for battery technology. These methods have been less utilised in this field but present the advantage of being able to achieve conformal coverage (even on high aspect-ratio substrates) and cheaper scale up in an industrial process [79,80]. In traditional CVD methods, the reagent gaseous precursors become homogeneously mixed before entering the reactor, while combinatorial CVD implements separate points of entry. The gradient in gas mixtures across the reactor induces compositional film growth, producing a single film with numerous phases and compositions, which can yield optimised functional properties and help to discover new materials [79]. In this way, the composites presented in Table 2 could be grown by a combinatorial CVD route-based multilayer approach involving the active materials in varying electrode proportions, making the whole process simple, since it would take place in one step and as a consequence be potentially very industrially competitive. Another advantage of the particular route is the optimisation of active materials morphology and particle size that could reduce the interface resistance among the electrode and electrolyte.

Special attention needs also to be paid at interfaces that exist between the electrode materials and electrolyte, between the electrodes and current collector, between different materials components within an electrode, etc. High-quality data from multiple approaches including experiments, testing, and modeling are required to accelerate the rational design of battery materials and interfaces. From that perspective, electrochemical analysis accompanied by for instance Raman spectroscopy to evaluate the structural changes of the electrode in various states will provide a deep understanding of the processes taking place in the interfaces. CFD studies on materials properties and interactions in numerous tests and environments could also be performed to maintain a sustainable design that will uphold robust properties and the optimum performance of the electrode per surface area in large scale for mass industrialisation. Hence, the development of both theory and experiment is a challenging way of producing the next generation of battery technologies avoiding or minimising trial and error routes.

Currently, the cell design is limited to some standard formats including cylindrical and pouch. The acceleration of new cell designs is essential to connect the desired properties related with the cell configuration, electrode compositions, and structures. This can be achieved through modeling opening up possibilities to explore new cell formats. On this point, we should not forget to include eco-design criteria allowing the recycling of parts or materials. While this is essential for future battery technology, a discussion of these factors is beyond the scope of the work presented here.

## 4. Conclusions

This review paper presents the progress made and challenges remaining for the fabrication of large-area electrode materials via a technology that is proven to be industrially competitive: that of chemical vapour deposition. This manufacturing process has both pros and cons but is shown to be capable of producing commercially viable novel electrode materials with high safety, performance, and cost reduction.

As has been shown, the fabrication of electrode materials by CVD for systems other than Li^+^ has not been widely investigated. Studies on multivalent ions such as Mg^2+^, Zn^2+^, and Al^3+^ are actually still in their infancy. From that perspective, it is equally important to test the electrodes in cells and evaluate their performance. It is increasingly challenging for these ions to reach high specific capacity and stability with the potential to act as inexpensive and efficient electrochemical devices.

Utilising the CVD route for the full implementation of the battery cell will save both time and money. New manufacturing designs through the computational simulation of the process constitute priorities to predict the optimised processing parameters and interfaces with the desired performance goals.

It is believed that the progress in electrode materials innovation taking into account the safety, low cost, and large scale along with high performance will encourage the leading role of aqueous batteries. Despite the fact that Li^+^ batteries are the dominating power source, the progress in post-Li^+^ batteries towards a more cost-effective and safer alternative will be strengthened in the coming years.

## Figures and Tables

**Table 1:** Ionic radius of metal electrodes [50].

Metal Electrode	Ionic Radius (Å)
Li	0.76
Na	1.02
K	1.38
Mg	0.72
Al	0.53
Zn	0.75

**Table 2:** A synopsis of the aqueous batteries utilising CVD processes.

Electrodes	CVD Approach	Electrolytes	Specific Capacity	Stability
SiO/graphite@C (examined in CR2032-type coin-type half batteries) [56]	FTCVD to cover SiO by the C layer	LBC3401A4	852.6 mAh g^−1^	93.7% capacity retention after 100 cycles
Monoclinic and metastable VO_2_ [60]	APCVD	1 M, LiOH	425 mAh g^−1^	97 % capacity retention after 500 cycles
Metastable VO_2_ [62]	APCVD	1 M, LiCl	505 mAh g^−1^	96% capacity retention after 500 cycles
α-axis textured monoclinic VO_2_ [63]	APCVD	1 M, LiOH	459 mAh g^−1^	97% capacity retention after 1000 cycles
Silver vanadate bronze [66]	AACVD	1 M, LiCl	230 mAh g^−1^	96% after 500 cycles
NaTi_2_(PO_4_)_3_ [70]	CVD to coat C on NaTi_2_(PO_4_)_3_	1 M, Na_2_SO_4_	80 mAh g^−1^	85% capacity retention after 200 cycles
Orthorhombic V_2_O_5_ [73]	AACVD	0.075 M, MgCl_2_	427 mAh g^−1^	82% capacity retention after 2000 cycles
Orthorhombic and monoclinic V_2_O_5_ [75]	AACVD	0.075 M, MgCl_2_	300 mAh g^−1^	92% capacity retention after 10,000 cycles
CNT/MnO_2_/PEDOT (examined in quasi solid-state battery device) [77]	CVD3D CNT	2 M, ZnCl_2_ and 0.4 M, MnSO_4_	306.1 mAh g^−1^	81.3% capacity retention after 2000 cycles
3D graphitic-foam (examined in Swagelok or pouch cells) [78]	CVD graphitic foam	AlCl_3_/1-ethyl-3-methylimidazolium (residual water ~500 p.p.m.	70 mAh g^−1^	~10% capacity retention after 7500 cycles

## Data Availability

The data presented in this study are available on request from the corresponding author.
